# In Vitro-Transcribed mRNA Chimeric Antigen Receptor T Cell (IVT mRNA CAR T) Therapy in Hematologic and Solid Tumor Management: A Preclinical Update

**DOI:** 10.3390/ijms21186514

**Published:** 2020-09-06

**Authors:** Thangavelu Soundara Rajan, Agnese Gugliandolo, Placido Bramanti, Emanuela Mazzon

**Affiliations:** 1 Department of Biotechnology, School of Life Sciences, Karpagam Academy of Higher Education, Coimbatore 641021, India; tsrajanpillai@gmail.com; 2 Karpagam Cancer Research Centre, Karpagam Academy of Higher Education, Coimbatore 641021, India; 3 IRCCS Centro Neurolesi “Bonino-Pulejo”, Via Palermo, Contrada Casazza–S.S.113, 98124 Messina, Italy; agnese.gugliandolo@irccsme.it (A.G.); placido.bramanti@irccsme.it (P.B.)

**Keywords:** adoptive T cell immunotherapy, chimeric antigen receptor, in vitro-transcribed mRNA, T cells, hematologic tumors, solid tumors

## Abstract

Adoptive T cell immunotherapy has received considerable interest in the treatment of cancer. In recent years, chimeric antigen receptor T cell (CAR T) therapy has emerged as a promising therapy in cancer treatment. In CAR T therapy, T cells from the patients are collected, reprogrammed genetically against tumor antigens, and reintroduced into the patients to trigger an immense immune response against cancer cells. CAR T therapy is successful in hematologic malignancies; however, in solid tumors, CAR T therapy faces multiple challenges, including the on-target off-tumor phenomenon, as most of the tumor-associated antigens are expressed in normal cells as well. Consequently, a transient in vitro-transcribed anti-mRNA-based CAR T cell (IVT mRNA CAR T) approach has been investigated to produce controlled cytotoxicity for a limited duration to avoid any undesirable effects in patients. In vitro and in vivo studies demonstrated the therapeutic ability of mRNA-engineered T cells in solid tumors, including melanoma, neuroblastoma and ovarian cancer; however, very few clinical trials are registered. In the present review, we discuss the effect of IVT mRNA CAR T therapy in preclinical studies related to hematologic malignancies and solid tumor management. In addition, we discuss the clinical trial studies based on IVT mRNA CAR T therapy in cancer.

## 1. Introduction

In recent years, adoptive T cell immunotherapy has emerged as a promising therapy for cancer patients. It is based on two methods: (i) to isolate the tumor-infiltrating lymphocytes from the primary tumor tissues of patients [1] and (ii) to construct T cells with defined specificity against tumor antigens using gene modification approaches [2]. Two gene modification approaches have been used to manufacture the monoclonal T cells with predetermined antigen specificity, namely T cell receptor (TCR) gene transfer and chimeric antigen receptor (CAR) gene transfer [2]. CAR T (CAR T) cells have received significant attention as the most promising adoptive immunotherapy for cancer. CAR T cells are genetically reprogrammed to express an antigen-specific, non-MHC-restricted receptor. This receptor is composed of the extracellular antigen recognition domain, which is most commonly derived from the single-chain variable fragment (scFv) of a monoclonal antibody fused to a hinge, a transmembrane domain, an intracellular signaling domain and/or co-stimulatory molecules [3,4]. Transformed CAR T cells are constructed using a plasmid or viral vector. 

**Figure 1:**
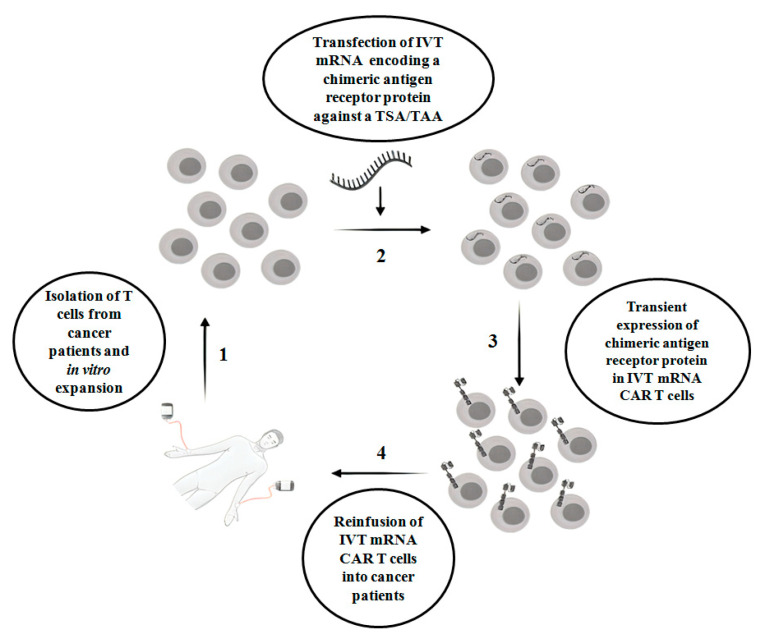
The scheme of IVT mRNA CAR T therapy in cancer patients. IVT mRNA CAR T: in vitro transcribed mRNA chimeric antigen receptor T cells; TSA: Tumor-specific antigen; TAA: Tumor-associated antigen.

CAR T therapy is successful in hematologic malignancies, for example, B cell malignancies, which include acute lymphoblastic leukemia, chronic lymphoblastic leukemia and non-Hodgkin lymphoma [5]. However, in solid tumors, CAR T therapy faces multiple challenges, with limited success. For example, unlike hematologic malignancies, finding an ideal single target antigen is more difficult in solid tumors. On the other hand, it is more common to detect a tumor-associated antigen(s) (TAA) in a solid tumor. TAAs are overexpressed in tumors but also expressed at the physiological level in normal non-tumor tissues. Proteins such as epidermal growth factor receptor (EGFR), carcinoembryonic antigen (CEA), epidermal growth factor receptor 2 (ERBB2), prostate-specific membrane antigen (PSMA) and mesothelin are examples of frequently targeted TAAs present in solid tumors [6]. Indeed, a lack of tumor antigen specificity of CAR T cells enhances the risk of substantial on-target off-tumor toxicity in normal tissues, which occurs when the indefinite period of CAR expression in T cells attacks non-tumor cells that display the intended antigens. This is one of the clinical challenges in the conventional CAR T therapy in cancer treatment. Other challenges include a lack of knowledge of appropriate tumor specific antigen (s) (TSAs)/TAAs, heterogeneity of tumor antigens, difficulties of CAR T cells to enter into tumor sites and the negative effect of the tumor microenvironment on CAR T cells [7]. In order to circumvent on-target off-tumor toxicity, in vitro transcribed mRNA CAR T (IVT mRNA CAR T) cells are emerging as a safe therapeutic approach, where T cells are transiently reprogrammed with mRNA that encodes chimeric membrane antigen receptor protein against a TSA or TAA. Due to the labile nature of mRNA, IVT- mRNA CAR T reduces the side effects associated with on-target off-tumor toxicity [8]. However, there are limitations associated with the IVT mRNA approach, which include a lack of sufficient longevity of mRNA-redirected T cells, which results in the expression of encoded protein for a few days, poor tumor infiltration, manufacturing challenges when a limited quantity of T cells are available and the risk of side effects when repeated doses of CAR T cells are injected. The scheme of IVT mRNA CAR T therapy in cancer patients is shown in Figure 1.

In the present review, we discuss the preclinical reports on the effect of IVT mRNA CAR T in hematologic and solid malignancies. Furthermore, we discuss the clinical trial studies based on IVT mRNA CAR T therapy in cancer. In order to collect research articles related to IVT mRNA CAR T therapy, we performed PubMed and Google Scholar searches using the following key words: “In vitro transcribed mRNA chimeric antigen receptor T cells and cancer”, “IVT- mRNA CAR T and cancer”, “In vitro mRNA CAR T and cancer”, “In vitro mRNA CAR T and hematologic tumors”, “In vitro mRNA CAR T and leukemia”, “In vitro mRNA CAR T and lymphoma”, “In vitro mRNA CAR T and solid tumors”, and “In vitro mRNA CAR T and cancer and case reports”.

## 2. Effect of IVT mRNA CAR T Cells in Preclinical Studies Related to Hematologic Malignancies 

### 2.1. Chronic Lymphocytic Leukemia (CLL)

CLL is a type of cancer, characterized by excessive production of lymphocytes by bone marrow in a slow progressive manner [9]. The CD19 surface antigen marker has been investigated in many studies of IVT mRNA CAR T against CLL. In a primitive in vitro study, Rabinovich et al. demonstrated the cytotoxic efficacy of human chimeric immune receptor T cells modified with mRNA which targeted B cell surface antigen CD19. This antigen is expressed in normal B cells and B cell-derived malignancies [10]. Moreover, the same group reported the killing ability of CD19-targeted mRNA-expressing human CD3+CD8+ cells in a humanized mouse model of Daudi lymphoma [11]. In another study, CD19-targeting mRNA in T lymphocytes collected from leukemia and lymphoma patients were investigated against B cell chronic lymphocytic leukemia (B-CLL) and mantle cell lymphoma (MCL) cells [12]. Degranulation and secretion of IFN-γ were noticed upon the recognition of CD19 in B-CLL and MCL cells. Moreover, in this study, CXCR4 or CCR7 mRNA was co-transfected together with CD19 mRNA to enhance the chemotactic response of the CAR T cells. Of note, in certain cancers, T cells may not reach the tumor site. Accordingly, insertion of CXCR4 or CCR7 mRNA contributes to the homing of CAR T cells towards bone marrow and lymphnodes, respectively, and binding with CD19 surface antigen. Köksal et al. demonstrated the killing efficiency and/or tumor growth in vitro and in vivo of CD37-targeting mRNA CAR T cells against human Burkitt’s lymphoma cell line BL41 and diffuse large B cell lymphoma cell line U-2932 [13]. In addition to murine models, the feasibility and safety of IVT mRNA CAR T cells has been investigated in canine models. Canine CD20-ζ mRNA CAR T cells induced modest antitumor activity in a dog with relapsed B cell lymphoma [14].

### 2.2. Acute Lymphoblastic Leukemia (ALL)

ALL is a type of cancer where the rapid progression of lymphocyte multiplication occurs in bone marrow. It is the most common childhood cancer [9]. As in CLL, CD19 is the most investigated tumor antigen for IVT mRNA CAR T cells versus ALL. Barrett et al. showed the cytotoxic potential of human CD19 mRNA-redirected T cells against the Nalm-6 human ALL cell line and K562 human erythroleukemic cell line engineered to express the CD19 antigen [15]. In the same study, similar killing potential of CD19-targeted CAR T cells was observed in a humanized mouse ALL model generated by Nalm-6 xenografting. In 2013, the same group examined the impact of regimen-based repeated infusions of CD19-targeted mRNA CAR T cells in a mouse Nalm-6–ALL xenograft model [16]. They suggested that the split dosage of mRNA-engineered CAR T cells and the timing of lymphodepletion serve a major role in enhancing the degree of the anti-leukemic effect of CD19-targeted mRNA CAR T cells. 

Almåsbak et al. documented the possible role of the non-signaling constituents present in CAR constructs [17]. They observed that the presence of a IgG1-CH2CH3 spacer in the CD19-targeted mRNA CAR construct inhibited the cytotoxic efficacy of the reprogrammed CAR T cells in a mouse Nalm-6–ALL xenograft model. Interestingly, the authors found CD19-independent severe toxicity in ALL mice. Further analyses revealed that the observed CD19-independent toxicity was attributed to the binding of the IgG1-CH2 spacer domain (which contains fragment c gamma receptor (FcγR)-binding motifs) of the CAR construct with soluble FcγR-1 of the macrophages of ALL mice, which caused off-target CAR T cell activation towards the mouse macrophages. Findings from this study highlighted the importance of the spacer in CAR constructs in clinical settings. Although these preclinical results seem to discourage the use of an IgG-derived hinge, the impact in a human clinical setting requires further investigation [18,19].

### 2.3. Acute Myeloid Leukemia (AML)

AML is a type of cancer with the rapid progression of myeloid cell multiplication in bone marrow [9]. CD33 and CD123 are the surface markers investigated for IVT mRNA CAR T cells in AML treatment [20]. Kenderian et al. designed CAR T cells with CD33-targeting mRNA and investigated their cytotoxic ability in in vitro and in vivo models of AML [21]. Specific cell death and loss of leukemia burden were noticed in the IVT CAR CD33 mRNA-treated human MOLM14 cell line and in a humanized mouse AML model xenografted with MOLM14 cells, respectively. In this study, CD33 mRNA-reprogrammed CAR T cells were administered with cyclophosphamide, an immunosuppressant drug, which augmented the persistence of the CAR T cells. In 2017, the same group documented the cytotoxic effect of CD123-targeted mRNA-engineered human CAR T cells in a humanized mouse AML model [22].

An overview of the results obtained in preclinical studies related to hematologic malignancies is shown in Table 1.

## 3. Effect of IVT mRNA CAR T Cells in Preclinical Studies Related to Solid Malignancies 

### 3.1. Mesothelioma and Colon Cancers

Mesothelioma is a type of malignant tumor that occurs in the tissues that line the heart, stomach or lungs. Mesothelin and fibroblast activation protein are the two identified TAAs in mesothelioma and malignant pleural mesothelioma [24], in which mesothelin is a widely studied antigen in IVT mRNA CAR T. Colon cancer is a type of malignant tumor occurring in the inner wall of the large intestine. TAAs identified in colon and colorectal cancer include human epidermal growth factor receptor 2 (HER2), CEA, epithelial cell adhesion molecule (EpCAM) and human leukocyte antigen (HLA) [25]. Zhao et al. reported that repeated administration (intratumor) of mesothelin-targeting IVT mRNA CAR T cells markedly reduced flank mesothelioma tumors in a mouse model [26]. Moreover, the authors found similar protection in a mouse model of disseminated intraperitoneal tumors obtained from a malignant mesothelioma patient after injecting mesothelin-targeting IVT mRNA CAR T cells derived from the same patient, which suggested that autologous T cells may be redirected against TAAs through IVT mRNA. 

Lehner et al. documented the cytolytic ability of mRNA-directed CAR T cells against natural killer group 2D receptor (NKG2D) in Ewing’s sarcoma family of tumors (ESFT) cell lines [27]. In this study, the authors observed that the expression of mRNA-coded NKG2D receptor lasted for few hours after transfection, while its expression was irreversibly reduced several hours after transfection, suggesting the controlled regulation of IVT NKG2D mRNA expression in T cells. 

### 3.2. Ovarian and Breast Cancers

Ovarian and breast cancers are common in older women. TAAs identified in ovarian cancer include HER2, hepatocyte growth factor receptor (c-Met), mesothelin, folate receptor alpha (FRα), cancer/testis antigen 1B and cancer antigen 125 [24]. TAAs identified in breast cancer include HER2, CEA, hepatocyte growth factor receptor (c-Met), NKG2D and ErbB2+MUC1 [24,28]. In 2009, Yoon et al. documented the evidence that human peripheral blood lymphocytes (PBLs) transfected with mRNA against receptor tyrosine protein kinase Her-2/neu killed human ovarian cancer cell line SKOV3 in vitro [29]. The cell death was attributed to the secretion of type I proinflammatory cytokines such as IFN-γ, IL 8 and granulocyte–macrophage colony stimulating factor (GM-CSF), chiefly mediated by CD8+ T cells. The authors coined the receptor-inserted PBL as chimeric immune receptor-PBL. Moreover, a significant reduction in tumor growth was found in an in vivo model of a SKOV3 cell-induced humanized mice xenograft model administered with chimeric immune receptor mRNA. Schutsky et al. demonstrated the anti-cancer ability of IVT mRNA CAR T cells against FRα, a protein that regulates cell growth, in ovarian cancer models [30]. In in vitro systems, human FRα-directed IVT mRNA CAR T cells killed human ovarian cancer cell lines OVCAR3, A187 and SKOV3. In addition, FRα-targeted mRNA significantly inhibited the cancer cell growth in localized and disseminated murine models of ovarian cancer.

In another study, human mesothelin-targeted mRNA was introduced in human peripheral lymphocytes and investigated in human in vitro and murine in vivo cancer models, with designed to express human mesothelin [31]. The results showed that mesothelin-targeted IVT CAR lymphocytes exerted cytotoxicity in the human K562 erythroleukaemia cell line and in the murine ovarian cancer Defb29/Vegf-a-luc cell line. Moreover, mesothelin-targeted IVT CAR lymphocytes significantly inhibited tumor growth in an in vivo humanized murine ovarian cancer model engineered to express human mesothelin. Anti-EpCAM mRNA CAR T cells produced anti-cancer activity in murine xenograft models of peritoneal ovarian and colorectal cancers by delaying the progression of tumor growth [32]. Hepatocyte growth factor receptor (c-Met) is a widely expressed TAA in solid tumors. In a recent study, it was reported that c-Met IVT mRNA-expressing T cells suppressed tumor growth in a murine ovarian cancer model and elicited a marked cytotoxic effect in human breast cancer cell lines BT20 and TB129 [33].

### 3.3. Neuroblastoma and Glioblastoma Multiforme

Neuroblastoma and glioblastoma multiforme are the most common malignant tumors arising from the central nervous system. TAAs identified in neuroblastoma include disialoganglioside GD2 and L1 cell adhesion molecule (L1-CAM). TAAs identified in glioblastoma multiforme include variant III of the epidermal growth factor receptor, HER2, CD133 and B7-H3 [24,34]. Selective antigen-targeted mRNA-incorporated CAR lymphocytes significantly modulate tumor growth in neuroblastoma models. Disialoganglioside GD2 is a potential tumor antigen expressed in human melanoma and neuroblastoma. Human GD2-targeted mRNA CAR T cells produced an anti-cancer effect in localized and disseminated murine neuroblastoma cancer models [35]. Intra-tumoral injection of GD2-IVT mRNA-targeted T cells rapidly killed the tumor cells; however, it was noticed that GD2 mRNA-targeted T cells were unable to reach the tumor site by intravenous injection and that multiple doses of GD2 mRNA-targeted T cells delayed only the tumor outgrowth and did not remove the disseminated neuroblastoma cells, suggesting the importance of preserving CAR surface expression on the periphery until reaching the tumor site and eliciting a cytotoxic response.

Caruso et al. demonstrated the cytolytic efficacy of anti-EGFR-targeted mRNA CAR T cells (human) against human glioblastoma cell lines U87, T98G and LN18 [36]. 

### 3.4. Melanoma

Melanoma is a malignant form of skin cancer. TAAs identified in melanoma include vascular endothelial growth factor receptor 2 (VEGFR2), gp100, TRP-1, TRP-2, GD2, L1-CAM, cancer/testis antigen 1B and melanoma-associated chondroitin sulfate proteoglycan (MCSP; also referred to as chondroitin sulfate proteoglycan 4 (CSPG4)) [24,37]. Human T cells constructed with IVT mRNA against MCSP revealed considerable antitumor effects against in vitro A375M and Melur melanoma cell lines and in an in vivo murine melanoma model [38]. The study also reported antigen-specific elevated proinflammatory cytokines in MCSP IVT mRNA CAR T cells in response to tumor cells. Similar anti-melanoma cancer activity was exerted by human T cells, including peripheral blood mononuclear cells, CD8+ T cells and γ/δ T cells, transfected with mRNA specific to either gp100/HLA-A2-TCRs or MCSP CARs against human melanoma cell lines A375M and Mel526, and the lymphoma cell line Daudi [39]. Among other T cells, γ/δ T cells exerted cytotoxicity against melanoma cell lines despite secreting a low level of cytokines compared to other T cells investigated in the study, suggesting that γ/δ T cells are a promising and potentially safer T cell population to be used in CAR T therapy for melanoma. 

In another study, human T cells were designed to express both gp100 TCRs and MCSP CARs using mRNA transfection and tested against A375M and Mel526 cell lines [40]. The authors of the study coined those T cells as T cells expressing two additional receptors (TETARs). Antigen-specific synthesis of IFN-γ, TNF and IL-2 cytokines were noticed in TETARS, which induced cell death in melanoma cell lines. In continuation of the above study, the same group constructed TETARs using combined DNA-based TCRs for gp100 (via lentiviral transduction) and mRNA-based CARs against CSPG4 and investigated the antitumor effect in the A375M cell line [41]. Both TCRs and CARs were expressed in T cells which killed A375M cells by antigen-based proinflammatory cytokines. Clinical-scale production of mRNA IVT CAR T cells redirected against melanoma-targeted CSPG4 was reported [42]. Moreover, the same group studied the efficacy of mRNA CARs expressed in natural killer T (NKT) cells against a melanoma cell line [43]. CSPG4-directed mRNA CARs were transfected in human NKT cells and cytotoxicity was evaluated in the A375M cell line. The results showed that CSPG4 CAR-directed NKT cells secreted proinflammatory IL-2, TNF-α and IFN-γ cytokines after recognizing specific antigens in the A375M cell line and thus induced cell death. Walseng et al. manufactured TCR-based CAR T cells and NK cell line NK-92 and studied the potential of therapeutic TCRs in NK cells [44]. It is imperative to mention here that unlike CARs, which are limited to detecting the surface antigens, TCRs can also recognize intracellular epitopes present in major histocompatibility complex (MHC) molecules. However, TCR activation depends on the signaling proteins present in the CD3 complex, which is restricted to T cells. Consequently, there might be competition between exogenous and endogenous TCRs to utilize the signaling proteins. Moreover, mispairing between TCRs may occur. In order to minimize these risks, a TCR-CAR construct was made by linking a single-chain soluble TCR with the transmembrane and signaling domains of a CAR construct. The results showed that the IVT mRNA TCR-CAR construct was able to conserve the specificity of the original TCR and was independent of the signaling molecules of the endogenous TCR. Moreover, a TCR-CAR-redirected NK-92 cell line induced cytotoxicity in tumor cells, suggesting the possibility to manufacture NK cells with the heterogenous target recognition of TCRs.

Inoo et al. reported the antitumor effect of anti-VEGFR2 IVT mRNA CAR T cells in a murine melanoma model [45]. Antigen-specific cytokine release was noticed in anti-VEGFR2 mRNA CAR T cells, which triggered cytotoxic events in cancer cells and suppressed tumor growth. 

Taken together, a substantial number of preclinical studies have been reported in hematologic and solid malignancies with IVT mRNA CAR T.

An overview of the results obtained in preclinical studies related to solid malignancies is shown in Table 2. 

## 4. IVT mRNA-Based Clinical Trials in Hematologic and Solid Tumors 

Clinical trials using IVT mRNA CAR T for hematologic and solid tumors have been initiated worldwide. Trials with anti-mesothelin CARs and anti-cMet CARs against malignant pleural mesothelioma (NCT01355965), metastatic pancreatic ductal adenocarcinoma (NCT01897415) and metastatic triple-negative breast cancer (NCT01837602) have been completed. A few of the ongoing clinical trials are studying the therapeutic effect of anti-cMet CARs, anti-CD19 CARs and anti-CD20 CARs against malignant melanoma breast cancer (NCT03060356), Hodgkin’s lymphoma (NCT02624258), B cell leukemia B cell lymphoma (NCT03166878) and non-Hodgkin’s lymphoma B cell chronic lymphocyticeukemia (NCT02315118). In a pilot clinical trial, CAR T cells reprogrammed with mRNA that targeted CD123 were tested in relapsed/refractory acute myeloid leukemia patients [46]. Although the method was safe, no antitumor effect was elicited by CAR T cells. Moreover, the study reported a poor quality of T cells from the patients and a lack of persistence of administered CAR T cells. In 2018, Svoboda et al. reported the transient responses of CD19-targeted mRNA engineered T cells (CD19 CAR T) in patients with relapsed or refractory classical Hodgkin’s lymphoma (cHL). This lymphoma is characterized by scant CD19^−^Hodgkin and Reed-Sternberg (HRS) cells within an immunosuppressive tumor microenvironment which causes limitations for cellular therapies directly targeting antigens expressed on HRS cells [47]. In this trial, the CD19 antigen was targeted against CD19^+^ B cells present in the tumor microenvironment and putative circulating CD19^+^HRS cells, which may result in the disruption of the tumor microenvironment necessary for the survival of HRS cells. Four patients were administered CD19 CART cells and obtained transient responses; one patient achieved a complete response and one patient achieved a partial response. Two patients showed no responses and this resulted in progressive disease and stable disease outcomes. All of them were shown to have no severe toxicity owing to the transient expression of the inserted CAR mRNA of the T cells.

Phase I clinical trials have been initiated to investigate the efficacy of these IVT CAR T cells redirected for mesothelin (CARTmeso cells) in malignant pleural mesothelioma patients and other tumors that overexpress mesothelin [48]. During the trial, severe anaphylaxis was observed in one patient, which was attributed to the sudden rise in immunoglobulin G (IgG) after two initial injections. Anaphylaxis may also result from an IgE-driven immune response to foreign CAR moieties that degranulate mast cells [49] and that multiple doses of CAR T cells may contribute to anaphylaxis reactions [48]. Consequently, the dosage schema was modified, followed by the absence of anaphylaxis. In a follow-up study, two of these patients showed partial antitumor responses as evidenced by the presence of novel circulating antitumor antibodies and tumor regression with a lack of apparent on-target off-tumor side effects in normal cells [50]. In another phase I clinical trial with six pancreatic ductal adenocarcinoma patients, CARTmeso cell treatment resulted in an elevated level of circulating antibodies produced against protumor-related proteins such as B cell maturation antigen, Ras-related protein Rab-11B, signal-transducing adaptor protein 1, programmed cell death protein 1 (PD1), programmed death ligand 1 (PDL1), and transducin-like enhancer protein 3 in one patient [51]. Tchou et al. reported that no side effect was noticed in metastatic breast cancer patients who intratumorally received c-Met IVT mRNA CAR T cells in a phase 0 clinical trial. Interestingly, immunohistochemical analysis from excised tumors displayed a high degree of necrosis, suggesting the anti-cancer effect of c-Met IVT mRNA CAR T cells in breast cancer patients [33]. A few more clinical trials are ongoing with breast cancer and colorectal cancer patients; however, no clinical updates have been provided.

An overview of the results obtained in clinical trials related to solid and hematologic malignancies is shown in Table 3.

## 5. Future Directions

A considerable number of preclinical studies have reported the promising and safer therapeutic ability of IVT mRNA CAR T cells in hematologic and solid tumors. Given the promising preclinical results from IVT mRNA CAR T cells, more clinical trials are warranted to investigate the therapeutic efficacy of mRNA-redirected CAR T cells in different cancers. To achieve this translational part, additional preclinical studies are required to study the cytotoxic and tumor-reducing efficacies of new mRNA-engineered CAR constructs manufactured against novel protumor antigens and to elicit the antitumor response via intravenous injections. In addition, future studies are warranted to circumvent the lack of sustainable function and potency associated with IVT mRNA. Foster et al. purified mRNA using recent RNA technology by inserting a modified 1-methylpseudouridin nucleoside into the mRNA to avoid immune stimulation and purified it of possible double-stranded RNA contaminants, which may halt the translation, with the help of RNase III [23]. Human CAR T cells engineered with purified CD19 mRNA exerted a twofold increase in cytotoxicity towards the Nalm-6 cell line and a 100-fold inhibition of leukemia burden in humanized ALL mice with enhanced persistence. More animal studies designed to investigate the purity level of mRNA required before transfection and to study the potential of re-constructed mRNA in T cells against tumor-specific antigens together with cytokine stimulatory signals are warranted, which might increase the number of clinical trials on IVT mRNA CAR T therapy in hematologic and solid tumors in the future. Last, but not least, more studies should focus on improving the homing of mRNA CAR T cells into the tumor microenvironment by neutralizing the localized immunosuppressive cues.

## 6. Conclusions

IVT mRNA CAR T cell therapy, a form of adoptive T cell therapy, has received significant attention due to its ability to drive away the tumor immune response from the on-target off-tumor phenomenon in hematologic and solid tumors. Preclinical studies have demonstrated the efficacy of these transiently redirected T cells in different hematologic and solid tumor models. Selective antigen-specific proinflammatory cytokine secretion, cell death induction, tumor growth reduction and prolonged survival are the key results from in vitro and in vivo studies. Limited clinical trials have reported positive responses for IVT mRNA CAR T cell therapy in the management of hematologic and solid tumors. Additional clinical trials with large sets of patients are warranted to test the therapeutic efficacy of IVT mRNA CAR T cells in hematologic and solid malignancies.

## Figures and Tables

**Table 1:** Preclinical studies regarding the effects of IVT mRNA CAR T cells in hematologic malignancies (i.p., intraperitoneal; i.n., intranodal; i.v., intravenous).

Type of Study	Target	Dose and Administration	Tumor Type	Results	Reference
in vitro	CD19	-	Leukemia, lymphoma	Cytotoxicity	[10]
in vivo	CD19	Multiple doses 5 × 10^6^; i.p.	lymphoma	Tumor growth inhibition	[11]
in vitro	CD19	-	Leukemia, lymphoma	Degranulation and IFN-γ secretion	[12]
In vitro and in vivo	CD37	Multiple doses 10^7^; intratumorally	Lymphoma	Cytotoxicity; tumor growth reduction	[13]
in vivo	Canine CD20-ζ	2.4 ×10^8^, 5.4 × 10^7^, 1.1 × 10^8^ cells i.v.; 1.16 × 10^8^ cells i.n.	Lymphoma	Antitumor activity	[14]
In vitro and in vivo	CD19	5 × 10^6^, 1 × 10^7^, or 2.5 × 10^7^ cells; i.v.	Leukemia	Cytotoxicity	[15]
in vivo	CD19	Multiple doses 1 × 10^7^ or 2 × 10^7^, 5 × 10^6^ and 5 × 10^6^; i.v.	Acute lymphoblastic leukemia	Increased cytotoxicity with split doses	[16]
in vivo	CD19	20 × 10^6^ or 10 × 10^6^; i.v.	Acute lymphoblastic leukemia	Abrogation of CD19-based cytotoxicity due to the presence of IgG1-CH2CH3 spacer in CAR construct	[17]
in vivo	CD19	2 × 10^7^; i.v.	Acute lymphoblastic leukemia	Cytotoxicity	[23]
In vitro and in vivo	CD33	5 × 10^6^; i.v.	Acute myeloid leukemia	Cytotoxicity; tumor growth reduction	[21]
in vivo	CD123	1 × 10^7^; i.v.	Acute myeloid leukemia	Cytotoxicity	[22]

**Table 2:** Preclinical studies regarding the effects of IVTmRNA CAR T cells in solid malignancies (i.p., intraperitoneal; i.v., intravenous).

Type of Study	Target	Dose and Administration	Tumor Type	Results	Reference
in vivo	Mesothelin	10–15 × 10^6^; intratumorally	Mesothelioma	Tumor growth reduction	[26]
in vitro	NKG2D	-	Ewing’s sarcoma family of tumors	Short-lived expression of mRNA	[27]
in vitro and in vivo	Her2	5 × 10^6^; intratumorally or i.p.	Ovarian cancer	Cytotoxicity; tumor growth reduction	[29]
in vitro and in vivo	FRα	Multiple doses 10^7^; i.p.	Ovarian cancer	Tumor growth inhibition	[30]
in vitro and in vivo	Mesothelin	1 × 10^7^ or 1 × 10^8^; i.p.	Ovarian cancer	Tumor growth inhibition	[31]
in vivo	Epithelial cell adhesion molecule	1 × 10^7^; i.p.	Ovarian and colorectal cancer	Tumor growth inhibition	[32]
in vitro and in vivo	c-Met	2 × 10^7^; i.p.	Breast and ovarian cancer	Cytotoxicity; tumor growth inhibition	[33]
in vivo	Disialoganglioside GD2	5 × 10^6^; intratumorally	Neuroblastoma	Cytotoxicity	[35]
in vitro	EGFR	-	Glioblastoma	Cytotoxicity	[36]
in vitro and in vivo	MCSP		Melanoma	Cytotoxicity	[38]
in vitro	gp100/HLA-A2 or MCSP	-	Melanoma	Cytotoxicity	[39]
in vitro	gp100 and MCSP	-	Melanoma	Cytotoxicity	[40]
in vitro	gp100 and CSPG4	-	Melanoma	Cytotoxicity	[41]
in vitro	CSPG4	-	Melanoma	Cytotoxicity	[43]
in vivo	VEGFR2	5 × 10^6^; i.v.	Melanoma	Tumor growth reduction	[45]

**Table 3:** Clinical trials regarding the effects of IVT mRNA CAR T cells in hematologic and solid malignancies (i.v., intravenous).

Phase	National Clinical Trial (NCT) No.	Target	Dose and Administration	Tumor Type	Results	Reference
Early Phase 1	NCT02623582	CD123	3 or 6 doses 4 × 10^6^cells/kg; i.v.	Relapsed/refractory acute myeloid leukemia	Safe method; no antitumor effects	[46]
Early Phase 1	NCT02277522 (adult); NCT02624258 (pediatric)	CD19	6 doses in the range 7.46 × 10^5^–2.11 × 10^6^; i.v.	Hodgkin’s lymphoma	No severe toxicity	[47]
Phase 1	NCT01355965	Mesothelin	Cohort 1: 1 × 10^8^ and 1 × 10^9^; Extended cohort: 3 doses of 1 × 10^8^ cells followed by 3 doses of 1 × 10^9^; i.v.	Malignant pleural mesothelioma	Severe anaphylaxis in one patient; partial antitumor response	[48,50]
Phase 1	NCT01897415	Mesothelin	3 doses weekly 1–3 × 10^8^/m^2^; i.v.	Metastatic pancreatic ductal adenocarcinoma	Increased expression of antitumor antibodies	[51]
Phase 0	NCT01837602	c-Met	3 × 10^7^ or 3 × 10^8^; intratumoral	Metastatic breast cancer	Anti-cancer effects	[33]

## Abbreviations

CAR T	Chimeric antigen receptor T cell
IVT mRNA CAR T	in vitro transcribed anti-mRNA-based CAR T cells
TCR	T cell receptor
CAR	Chimeric antigen receptor
scFv	Single-chain variable fragment
TAA	Tumor-associated antigen
EGFR	Epidermal growth factor receptor
CEA	Carcinoembryonic antigen
ERBB2	Epidermal growth factor receptor 2
PSMA	Prostate-specific membrane antigen
B-CLL	B cell chronic lymphocytic leukemia
B-NHL	B cell non-Hodgkin’s lymphoma
FcγR	Fragment c gamma receptor
BiTEs	Bispecific T cell engager
AML	Acute myeloid leukemia
NKG2D	Natural killer group 2D
ESFT	Ewing’s sarcoma family of tumors
TGFβRIImut	Transforming growth factor β receptor II frameshift mutation
GM-CSF	Granulocyte–macrophage colony Stimulating factor
FRα	Folate receptor alpha
EpCAM	Epithelial cell adhesion molecule
GBM	Glioblastoma multiforme
gp100	Glycoprotein 100
TRP-1	Tyrosinase-related protein 1
TRP-2	Tyrosinase-related protein 2
GD2	Disialoganglioside 2
L1-CAM	L1 cell adhesion molecule
MCSP	Melanoma-associated chondroitin sulfate proteoglycan
TETARs	T cells expressing two additional receptors
CSPG4	Chondroitin sulfate proteoglycan 4
NKT	Natural killer T
VEGFR2	Vascular endothelial growth factor receptor 2
CARTmeso cells	CAR T cells redirected for mesothelin
IgG	Immunoglobulin G
PD1	Programmed cell death protein 1
PDL1	Programmed death ligand 1
